# Relationship between visible branch arteries distal to the stenosis on magnetic resonance angiography and stroke recurrence in patients with severe middle cerebral artery trunk stenosis: a one-year follow up study

**DOI:** 10.1186/s12883-015-0423-0

**Published:** 2015-09-16

**Authors:** Hongbing Chen, Zhuhao Li, Hua Hong, Shihui Xing, Gang Liu, Aiwu Zhang, Shuangquan Tan, Jian Zhang, Jinsheng Zeng

**Affiliations:** Department of Neurology and Stroke Center, The First Affiliated Hospital, Sun Yat-Sen University, No. 58 Zhongshan Road II, Guangzhou, 510080 P. R. China; Department of Radiology, The First Affiliated Hospital, Sun Yat-Sen University, No. 58 Zhongshan Road II, Guangzhou, 510080 P. R. China

## Abstract

**Background:**

To evaluate the relationship between the flow signal intensity of branch arteries distal to the stenosis on 3-dimensional (3D) time-of-flight (TOF) magnetic resonance angiography (MRA) and the risk of stroke recurrence in patients with severe middle cerebral artery (MCA) trunk stenosis.

**Methods:**

We prospectively recruited 153 patients (mean age 62.9 ± 13.0 years, 106 males) with a first ischemic stroke or transient ischemic attack caused by a severe MCA trunk stenosis (70 % to 99 %) confirmed by 3D TOF MRA and followed them for one year to determine the stroke recurrence. The MCA branch signal intensity distal to the site of stenosis on 3D TOF MRA was classified as either good (grade A) or poor [mild reduction (grade B) or severe reduction (grade C)] according to the extent to which the MCA could be visualized. The patients were divided into groups A (35), B (58), or C (60) based on the MRA grading of the MCA branch signal intensity distal to the site of stenosis.

**Results:**

Poor MCA branch signal intensity was associated with internal border-zone infarction (*p* < 0.05). The risk of stroke recurrence in the ipsilateral MCA in the first year was 18.3 %. The 1-year cumulative incidence of recurrence was higher in the patients in group C (30 %) than in groups B (12.1 %) or A (8.6 %) (Log rank, *p* = 0.007). Multivariate analyses via Cox proportional hazard regression demonstrated that only a grade C classification of the signal intensity of the MCA branches was an independent predictor of stroke recurrence in the ipsilateral MCA (hazard ratio = 3.0, 95 % confidence interval = 1.3-7.4, *p* = 0.014).

**Conclusions:**

This study demonstrated that MCA branch signal intensity as assessed via 3D TOF MRA may be a useful and simple tool to stratify the risk of stroke recurrence in patients with severe MCA trunk stenosis.

## Background

Intracranial atherosclerosis is the most common cause of stroke in Asian patients [1–4], and intracranial artery stenosis (ICAS) is associated with a high risk of recurrent stroke [5–7]. However, patients with a recent stroke or transient ischemic attack (TIA) due to severe ICAS (70 % to 99 %) have a particularly high risk of stroke recurrence in the stenotic artery (17.6 % to 23 % at 1 year) [6, 8, 9]. The major treatments of ICAS include antiplatelet drugs, statins, risk factor control, and endovascular revascularization [10]. The Stenting and Aggressive Medical Management for Preventing Recurrent Stroke in Intracranial Stenosis (SAMMPRIS) trial recruited only patients with symptomatic severe ICAS and demonstrated that aggressive medical therapy is superior to stenting. However, the patients in the medical arm of SAMMPRIS still showed a high risk of stroke in the first year (12.2 %) [11]. Developing a simple method to stratify the risk of stroke recurrence due to ICAS is very important for treatment decisions and selecting patients for clinical studies. The Warfarin Aspirin Symptomatic Intracranial Disease (WASID) study used percutaneous cerebral angiography to investigate ICAS and found that, for severe ICAS, more extensive collateral blood flow diminished the risk of subsequent territorial stroke and the risk of a future stroke increased linearly with the percentage of stenosis [7, 12]. Transcranial Doppler studies also showed that both micro-embolic signals and progressive stenosis lesions are associated with recurrent stroke in symptomatic middle cerebral artery (MCA) stenosis [13, 14]. However, the above methods are limited in clinical practice due to the potential risk of complications of invasive tests, the need for relevant experts and equipment, or a lack of timeliness.

Magnetic resonance angiography (MRA) has become a commonly employed non-invasive imaging modality used to detect intracranial stenosis, and has a good sensitivity for the detection and evaluation of ICAS [15–18]. Three-dimensional (3D) time-of-flight (TOF) MRA uses the signals generated by the inflow of fresh, unsaturated and fully magnetized blood spins into the slab [19, 20]. These spins are gradually saturated during movement within the slab, and this saturation effect could lead to signal intensity loss in the peripheral arteries. The signal intensity loss due to the saturation effect is flow velocity-dependent and is more pronounced with lower flow velocity [21], particularly in single-slab 3D TOF MRA [20]. Several recent studies have used the signal intensity ratio (SIR) across the stenosis lesion on MRA to assess the change in signal intensity across an ICAS and observed that a decrease in SIR may be associated with hemodynamic disorders and a high risk of stoke recurrence [22–24]. This method is relatively complex. For example, specialized staffs are needed to analyze the MRA images, and different institutions have to establish different cut-points of SIR to determine its clinical significance. However, there is a simple method of assessing the change in signal intensity distal to the stenosis on MRA using the visibility of branch arteries that are distal to the stenosis. Previous studies have demonstrated that the visibility of the MCA stem or branches on MRA is associated with the hemodynamic status in the cerebral hemisphere with ipsilateral internal carotid artery (ICA) occlusive diseases and that the visibility of the MCA ipsilateral to carotid endarterectomy (CEA) on preoperative MRA may be useful for identifying patients who are at risk for cerebral hyperperfusion or ischemic events after CEA [25–27].

The MCA is a common site of ICAS occurrence [28, 29]. The reduced visibility of the branch arteries distal to stenosis on MRA could also be observed in many patients with severe MCA stenosis, but its clinical significance is unclear. This study evaluated the relationship between the signal intensity of the MCA branches of branch arteries distal to the stenosis on 3D TOF MRA and the risk of stroke recurrence in patients with severe MCA trunk stenosis resulting in an initial ischemic stroke or TIA.

## Methods

### Patients

Among 2794 adult patients with a first-ever ischemic stroke or TIA who were admitted to our stroke center within 7 days of stroke onset between January 2009 and April 2014, there were 184 patients with an ischemic stroke or TIA in the unilateral MCA territory and an ipsilateral severe MCA trunk stenosis (70 % to 99 %) confirmed by 3D TOF MRA. An ischemic stroke was defined as a new focal neurological deficit of sudden onset that was associated with new acute infarctions on imaging. A TIA was defined as a new focal neurological deficit of sudden onset that completely reversed within 24 h, without new acute infarctions on diffusion-weighted imaging (DWI). Among these patients, we excluded 11 patients with significant ipsilateral intra or extracranial carotid stenosis (>50 %) or occlusion, 2 patients with cardioembolic risk (1 patient with atrial fibrillation and 1 patient with valvular heart disease), 5 patients whose etiologies of ipsilateral MCA stenosis were non-atherosclerotic diseases or could not be determined, 8 patients with severe stenosis (>70 %) or occlusion in the contralateral MCA or carotid artery, 3 patients who underwent cranial MRA at other medical institutions, and 2 patients with contraindications for antithrombotic drugs or statins. Thus, we prospectively included 153 patients with symptomatic severe atherosclerotic MCA trunk stenosis in this study. This study was approved by the ethics committee of The First Affiliated Hospital of Sun Yat-Sen University. All patients or their legal representatives provided written informed consent before being included in this study.

### Brain magnetic resonance imaging and angiography

Brain magnetic resonance imaging (MRI) and cranial MRA were performed within 7 days of stroke onset in all patients using a 3.0 T (Magnetom Trio Tim 3.0 T; Siemens) MRI unit with a circular head coil. The MRI parameters for T1-weghted imaging (repetition time [TR], 500 ms; and echo time [TE], 8.9 ms), T2-weighted imaging (TR, 4000 ms; and TE, 100 ms), fluid attenuated inversion recovery (TR, 9000 ms; TE, 111 ms; and inversion time, 2500 ms), and DWI (TR, 5800 ms; TE, 100 ms; matrix number, 384 × 384; and two *b* values of 0 and 1000 s/mm^2^) included a slice thickness of 6 mm, an interslice gap of 1.2 mm, 19 axial slices, and a field of view of 229 × 229 mm. We used previously published arterial supply templates to determine the topography of the acute infarction lesions in the vascular territories of the MCA [30], and the distributions of infarction lesions were classified into perforating artery infarctions, cortical infarctions, and internal border-zone infarctions [31, 32]. One author (Z.L.) blinded to the clinical and vascular data evaluated the MRI images and determined the distributions of infarction lesions based on the DWI results.

Cranial MRA images were obtained using a single slab placed perpendicular to the basilar artery and centered on the circle of Willis. The 3D TOF images were acquired with the following parameters: TR/TE, 21/3.6 ms; flip angle, 15°; field of view, 219 × 219 mm; matrix number, 768 × 768; and axial slice thickness, 0.7 mm. The degree of intracranial stenosis was determined using a published method [33]. On 3D TOF MRA, severe MCA stenosis was defined as a 70 % to 99 % reduction in diameter or a focal signal loss with presence of distal MCA signal [18].

**Fig. 1:**
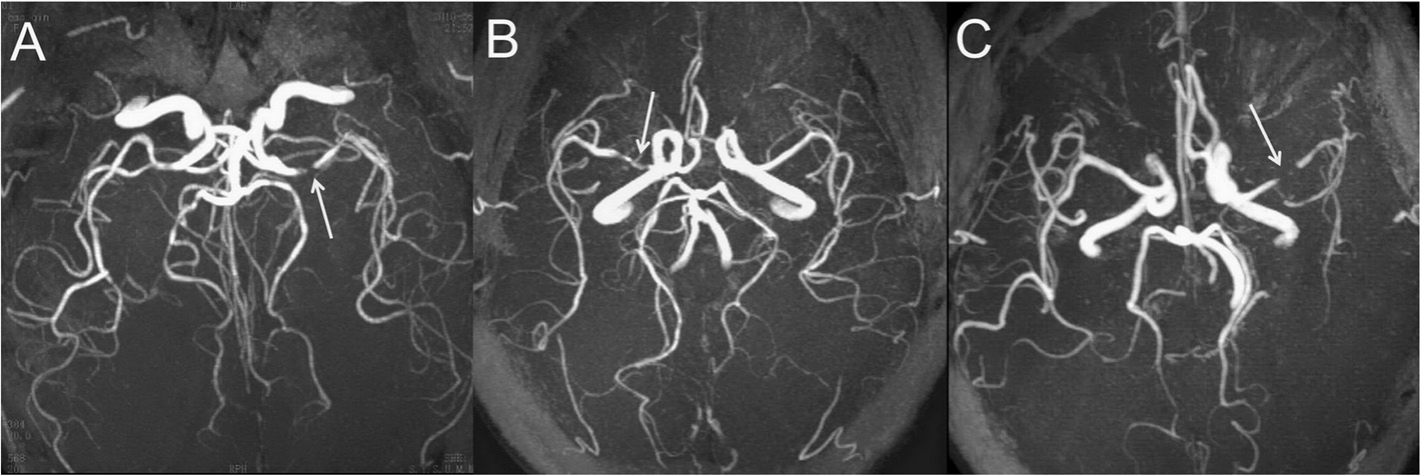
The grade of visualization of the MCA branches distal to the site of stenosis on MRA. **a**: grade A, the signal intensity of the MCA branches distal to the stenosis (white arrow) is normal or nearly symmetrical compared with the contralateral MCA; **b**: grade B, the signal intensity of the MCA branches distal to the stenosis (white arrow) is mildly reduced compared with the contralateral MCA, all M2 branches could be observed along its course, and two or more M3 branches could not be visualized to the cortical surface compared with the contralateral MCA or the number of visible M3 branches was at least 2 fewer than that of the contralateral MCA; and **c**: grade C, the signal intensity of the MCA branches distal to the stenosis (white arrow) is severely reduced compared with the contralateral MCA, and one or more M2 branches could not be visualized along its course compared with the contralateral MCA

We modified a method previously used to classify the grade of the signal intensity of the ipsilateral MCA on 3D TOF MRA in patients with ICA stenosis [25]. The signal intensity of the branch arteries distal to the site of the MCA trunk stenosis on 3D TOF MRA was visually classified as good [the signal intensity of the MCA branches was normal or nearly symmetric compared with the contralateral MCA (grade A)] or poor. A poor classification was divided into either a mild reduction: all M2 branches could be observed along its course, and two or more M3 branches could not be visualized to the cortical surface compared with the contralateral MCA or the number of visible M3 branches was at least two fewer than that of the contralateral MCA (grade B); or a severe reduction: one or more M2 branches could not be visualized along its course compared with the contralateral MCA (grade C) (Fig. 1).

Two authors (first, H.C.; and second, H.H.), who were blinded to the clinical and brain MRI data, independently investigated the images of 3D TOF MRA of all patients using an appropriate window width/level for displaying MCA branches on a picture archiving and communication system and determined the MRA grading of the signal intensity of the MCA branches distal to the site of stenosis. Disagreements were resolved by consensus. The patients were divided into three groups according to the grade of the signal intensity of the MCA branches. In addition, for assessing the intraobserver agreement of the MRA grading of MCA branches distal to stenosis, the first observer investigated the same images a second time after a three-month interval.

### Other diagnostic examinations

All patients underwent cervical vascular ultrasound. Cervical computed tomographic angiography (CTA) was performed in 33 patients and cervical MRA was performed in 61 patients according to clinical requirements. The degree of extracranial ICA stenosis was measured using the method used in the North American Symptomatic Carotid Endarterectomy Trial [34]. The cervical vascular ultrasound results were used for patients who did not undergo cervical CTA or MRA. We also investigated asymptomatic cerebrovascular atherosclerosis (atherosclerotic stenosis of >50 % or occlusion) of the major intra (using cranial MRA) and extracranial (using cervical CTA or MRA or the results of cervical vascular ultrasound in patients who did not undergo cervical CTA or MRA) arteries.

All patients underwent extensive blood tests, urine analysis, chest X-ray, 12-lead electrocardiogram, and transthoracic echocardiography. Additionally, 24-h electrocardiogram monitoring and transesophageal echocardiography were performed on selected patients according to clinical requirements.

### Clinical data

We prospectively recorded the following clinical data: age, sex, and risk factors. The risk factors for cerebrovascular disease included hypertension (previously diagnosed or patient using antihypertensive drugs before the stroke or blood pressure ≥ 140/90 mmHg during the nonacute phase), hypercholesterolemia (total cholesterol ≥ 5.7 mmol/L or low-density lipoprotein-cholesterol ≥ 3.6 mmol/L, measured within 48 h after admission or patient using lipid-lowering drugs before the stroke), diabetes mellitus (fasting glucose > 7 mmol/L during the nonacute phase or glycated hemoglobin > 6.5 %, measured during hospitalization or use of an antidiabetic therapy), and smoking (current or former habit of cigarette smoking). Patients’ clinical severity was assessed using the National Institute of Health Stroke Scale score within 6 h after admission to our center. Ischemic stroke subtypes were determined according to the criteria of the Trial of Org 10172 in Acute Stroke Treatment [35].

### Treatment and follow-up

All patients received antiplatelet (aspirin 100 mg/day or clopidogrel 75 mg/day) and statin (atorvastatin 40 mg/day) therapy at the time of discharge. We also managed the risk factors of cerebrovascular atherosclerosis (such as hypertension and diabetes). All patients were followed up at 3, 6, and 12 months after stroke onset via in-person or telephone interviews with the patients or their family members. A trained clinical research co-ordinator blinded to the patients’ clinical information conducted the interviews using a structured questionnaire. The stroke recurrence was further determined using the medical records of hospitalization or emergency. If needed, we contacted the hospital, where patients with a recurrent event were admitted, to obtain the detailed information regarding these recurrent events.

### Statistical analyses

Continuous variables were expressed as the means ± standard deviations. Independent-samples *t* test was used to compare continuous variables; Pearson’s test or Fisher’s exact test were used to compare categorical variables. The kappa test was used to analyze the MRA grading interobserver and intraobserver agreements. A Kaplan-Meier curve was generated for the cumulative probabilities of stroke recurrence in the region of the ipsilateral MCA. Cox proportional hazard regression for the univariate and multivariate analyses was used to identify potential predictors of recurrence. We selected the variables for inclusion in the multivariate regression analyses according the results of the univariate regression analyses (*p* ≤ 0.1). All statistical analyses were performed using PASW Statistics (version 18.0, IBM SPSS Statistics, Chicago, U.S.A.). Statistical significance was set at *p* < 0.05, and all tests were 2-sided.

## Results

There were 106 men and 47 women and the mean age was 62.9 ± 13.0 years (ranging from 38 to 82 years). Thirty-five (22.9 %), 58 (37.9 %), and 60 (39.2 %) patients were classified into groups A, B, and C, respectively. The interobserver (concordant rate = 0.93, κ = 0.890) and intraobserver (concordant rate = 0.94, κ = 0.910) agreements were excellent for the MRA grading of the signal intensity of branch arteries distal to the MCA stenosis. No significant differences in basic characteristics were observed between the patients with good signal intensity of the MCA branches and those with poor signal intensity of the MCA branches or between the patients of different groups (p > 0.05) (Table 1). For the distributions of infarction lesions in the patients present with ischemic stroke, internal border-zone infarctions were more common among the patients in group B and/or C than among the patients in group A (*p* < 0.05) (Table 2).

**Table 1:** Basic characteristics

	Total (*n* = 153)	Signal intensity distal to MCA stenosis				*P* Value (good vs poor)
		Good	Poor			
		Group A (*n* = 35)	Total (*n* = 118)	Group B (*n* = 58)	Group C (*n* = 60)	
Male	106(69.3)	24(68.6)	82(69.5)	43(74.1)	39(65)	0.917
Age^*^, years	62.9 ± 13.0	66.4 ± 11.9	61.9 ± 13.2	61.4 ± 13.4	62.4 ± 13.1	0.073
Cerebral infarction	144(94.1)	32(91.4)	112(94.9)	53(91.4)	59(98.3)	0.441
Transient ischemic attack	9(5.9)	3(8.6)	6(5.1)	5(8.6)	1(1.7)	
Risk factor						
Hypertension	106(69.3)	25(71.4)	81(68.6)	42(72.4)	39(65)	0.837
Hypercholesterolemia	75(49.0)	17(48.6)	58(49.2)	31(53.4)	27(45)	1.000
Diabetes	62(40.5)	13(37.1)	49(41.5)	24(41.4)	25(41.7)	0.698
Smoking	72(47.1)	17(48.6)	55(46.6)	28(48.3)	27(45)	0.838
NIHSS score at admission^*^	4.5 ± 3.5	4.1 ± 3.6	4.6 ± 3.5	4.1 ± 3.1	5.3 ± 3.9	0.435
Asymptomatic ICAS	74(48.4)	16(45.7)	58(49.2)	26(44.8)	32(53.3)	0.848
Asymptomatic ECAS	10(6.5)	3(8.6)	7(5.9)	4(6.9)	3(5)	0.579

*Abbreviations*: ECAS, exracranial atherosclerosis; ICAS, intracranial atherosclerosis; MCA, middle cerebral artery; NIHSS, National Institute of Health Stroke Scale

^*^Continuous variables expressed as the means ± standard deviations; other values are expressed as *n* (%)

**Table 2:** Distributions of infarction lesions in patients presented with ischemic stroke

	Total (*n* = 144)	Signal intensity distal to MCA stenosis				*P* Value (good vs poor)
		Good	Poor			
		Group A (*n* = 32)	Total (*n* = 112)	Group B (*n* = 53)	Group C (*n* = 59)	
Perforating artery infarctions	68(47.2)	18(56.3)	50(44.6)	25(47.2)	25(42.4)	0.316
Cortical infarctions	78(54.2)	14(43.8)	64(57.1)	32(60.4)	32(54.2)	0.228
Internal border-zone infarctions	80(55.6)	11(34.4)^*^	69(61.6)	31(58.5)	38(64.4)	0.008

*Abbreviations*: MCA, middle cerebral artery

All values are expressed as n (%)

^*^Group A versus group B and group C, *p* < 0.05

**Fig. 2:**
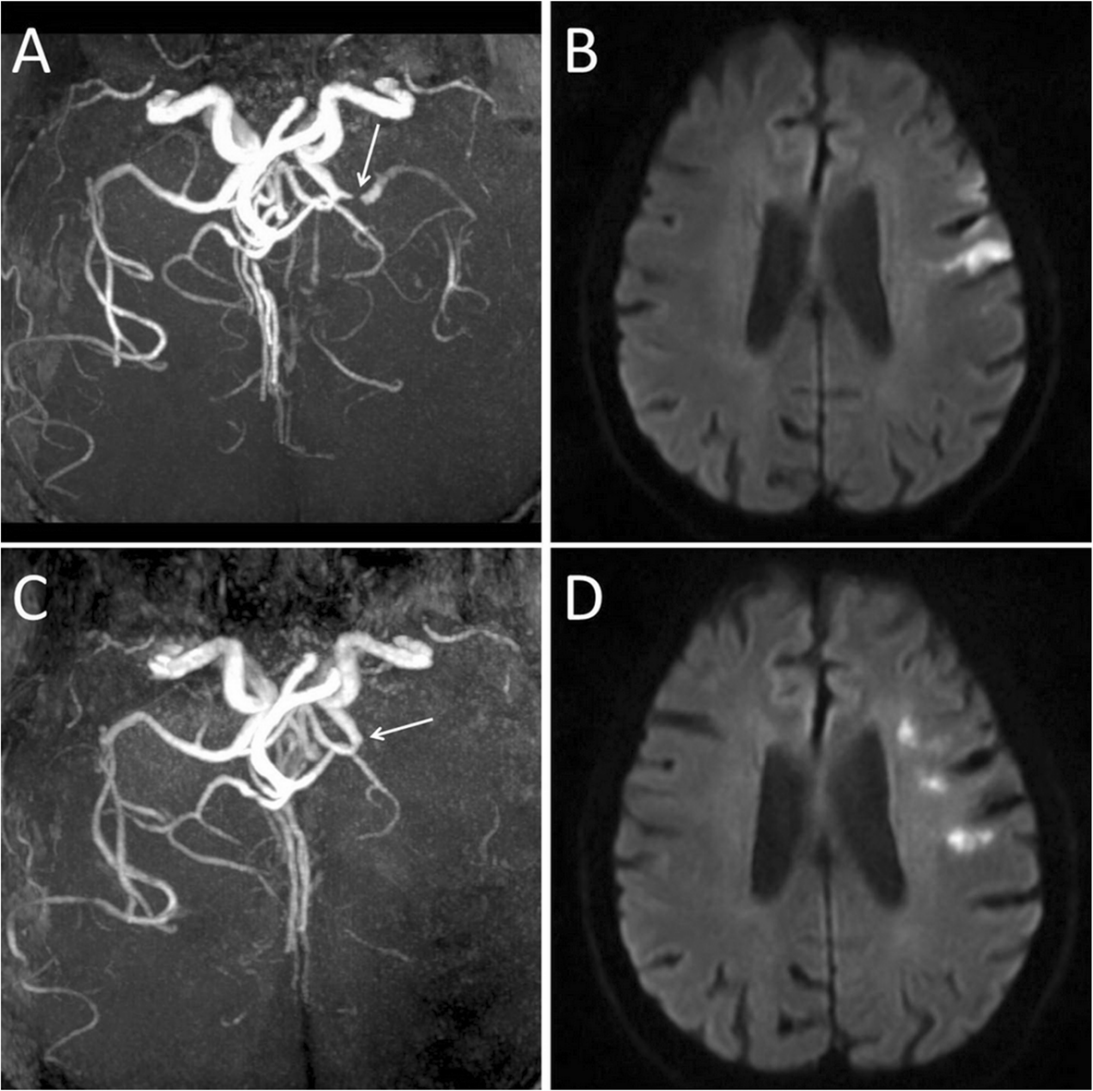
A case of stroke recurrence. On the third day after stroke onset, MRA (**a**) shows a severe stenosis of the left MCA trunk (white arrow) and a severely reduced signal intensity of the left MCA branches distal to the stenosis (grade C); DWI (**b**) shows acute cortical infarctions of the left frontal lobe. This patient experienced a recurrent stroke on the 71st day after the initial onset. On the 73rd day after the initial onset, MRA (**c**) shows a proximal occlusion of the left MCA trunk (white arrow); DWI (**d**) shows acute cortical and subcortical infarctions of the left frontal lobe

**Fig. 3:**
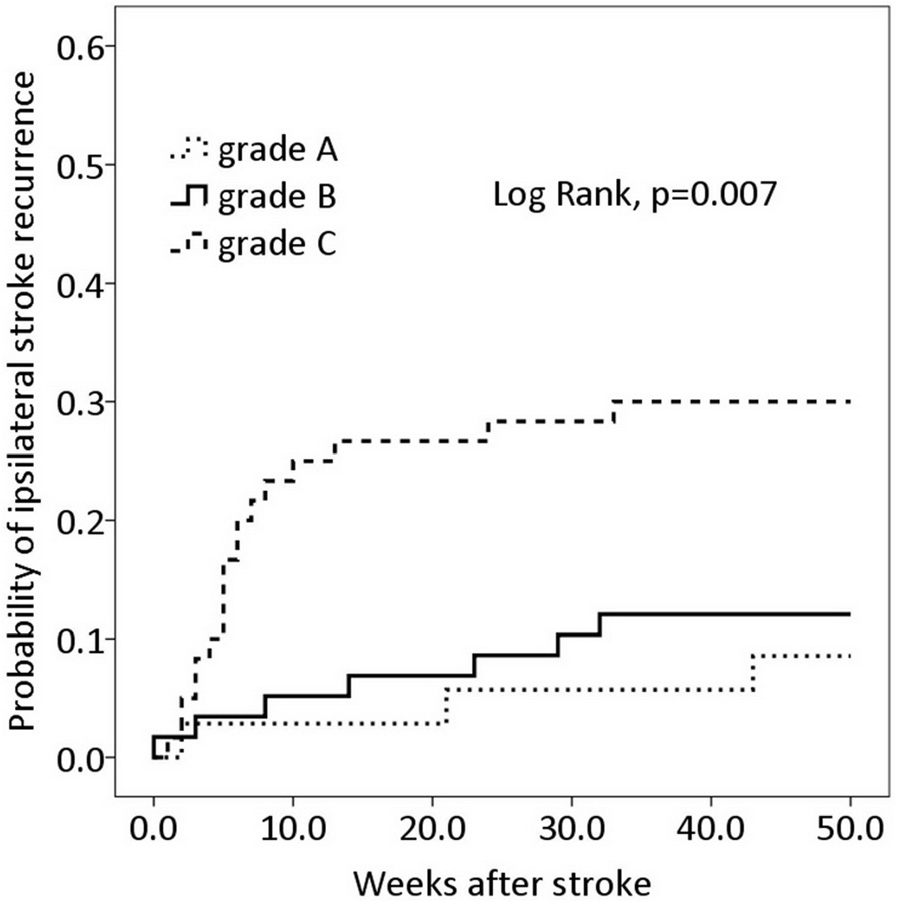
The Kaplan-Meier curves of ipsilateral stroke recurrence in patients with different grades of signal intensity of branch arteries distal to the MCA stenosis

All patients survived through their hospitalizations and follow-ups and completed 1-year follow-up. During the follow-up period, 2 patients in group A, 3 patients in group B, and 2 patients in group C discontinued their antiplatelet drugs for various reasons (*p* = 0.834); 4 patients in group A, 8 patients in group B, and 5 patients in group C discontinued statin use for various reasons (*p* = 0.639). Twenty-eight (18.3 %) patients experienced stroke recurrence in the ipsilateral MCA (23 ischemic strokes and 5 TIAs), which occurred from 0.5 to 43 weeks (median 6 weeks) after stroke onset, and 32 % and 64 % of the recurrences occurred within 4 and 12 weeks after stroke onset, respectively. Recurrence was observed in 3 (8.6 %) patients in group A, 7 (12.1 %) patients in group B, and 18 (30 %) patients in group C (Fig. 2). The Kaplan-Meier curve demonstrated that the cumulative incidence of recurrence was higher among the patients in group C than among the patients in groups A or B (Log rank, *p* = 0.007) (Fig. 3). Additionally, during the follow-up period, there were 3 patients who experienced stroke recurrence in areas other than the ipsilateral MCA (2 ischemic strokes and 1 TIA).

The univariate analyses of the Cox proportional hazard regression demonstrated that stroke recurrence in the ipsilateral MCA was associated only with a grade C classification of the signal intensity of the branch arteries distal to the MCA stenosis [hazard ratio (HR) = 3.2; 95 % confidence interval (CI) = 1.5-6.9; *p* = 0.003] (Table 3). In the multivariate analyses of the Cox proportional hazard regression adjusted for demographic and risk factors, a grade C classification of the signal intensity of the branch arteries distal to the MCA stenosis was the only independent predictor of stroke recurrence in the ipsilateral MCA (HR = 3.0; 95 % CI = 1.3-7.4, *p* = 0.014) (Table 4).

**Table 3:** Potential predictors of stroke recurrence in the ipsilateral MCA in univariate analyses

	Stroke recurrence, *n* (%)			Univariate analyses	
	Yes (*n* = 28)	No (*n* = 125)	*P* value	HR (95 % CI)	*P* value
Age ≥ 65 years	15(53.6)	58(46.4)	0.535	1.3(0.6-2.8)	0.445
Male	21(75)	85(68)	0.651	1.4(0.6-3.3)	0.459
Hypertension	22(78.6)	84(67.2)	0.267	1.7(0.7-4.1)	0.276
Hypercholesterolemia	12(42.9)	63(50.4)	0.534	0.8(0.4-1.6)	0.487
Diabetes mellitus	10(35.7)	52(41.6)	0.672	0.8(0.4-1.8)	0.603
Smoking	15(53.6)	57(45.6)	0.531	1.3(0.6-2.8)	0.438
Asymptomatic ICAS	15(53.6)	59(47.2)	0.676	1.3(0.6-2.7)	0.514
Distributions of cerebral infarctions					
Perforating artery infarctions	9(32.1)	59(47.2)	0.207	0.6(0.3-1.3)	0.168
Cortical infarctions	16(57.1)	62(49.6)	0.534	1.6(0.7-3.4)	0.241
Internal border-zone infarctions	16(57.1)	64(51.2)	0.677	1.3(0.6-2.8)	0.438
NIHSS score at admission ≥ 4	11(39.3)	62(49.6)	0.404	0.9(0.4-2.0)	0.855
Poor MCA branch signal intensity	25(89.3)	93(74.4)	0.134	2.8(0.8-9.3)	0.091
Grade C	18(64.3)	42(33.6)	0.005	3.2(1.5-6.9)	0.003
Discontinued antiplatelet drug	1(3.6)	6(4.8)	1.000	0.5(0.1-3.6)	0.481
Discontinued statin	4(14.3)	13(10.4)	0.554	1.2(0.4-3.1)	0.759

*Abbreviations*: CI, confidence interval; HR, hazard ratio; ICAS, intracranial atherosclerosis; MCA, middle cerebral artery; NIHSS, National Institute of Health Stroke Scale

**Table 4:** Potential predictors of stroke recurrence in the ipsilateral MCA in multivariate analyses

	HR (95 % CI)	*P* value
Age ≥ 65 years	1.2(0.5-2.7)	0.631
Male	1.5(0.5-4.0)	0.441
Hypertension	1.6(0.7-4.1)	0.368
Hypercholesterolemia	0.8(0.4-1.7)	0.544
Diabetes mellitus	0.8(0.4-1.8)	0.664
Smoking	1.4(0.6-3.3)	0.450
Poor MCA branch signal intensity	1.5(0.4-5.8)	0.561
Grade C	3.0(1.3-7.4)	0.014

*Abbreviations*: CI, confidence interval; HR, hazard ratio; MCA, middle cerebral artery

## Discussion

There was excellent interobserver and intraobserver reproducibility for the MRA signal intensity grading of the branch arteries distal to the MCA stenosis. We used a study period of one year because previous studies had demonstrated that most recurrent strokes in the territory of a symptomatic severe ICAS occurred during the first year after stroke onset in the setting of conventional treatment (12.2 %-23 % at the first year) and the risk of recurrence significantly declined after the first year [6, 11]. Our data demonstrated a high risk of stroke recurrence in the ipsilateral MCA in the first year after stroke onset in patients with ischemic stroke or TIA caused by severe MCA trunk stenosis (18.3 %), particularly in patients with severely reduced MCA branch signal intensity on MRA (30 %). The risk of recurrence in the first year in our patients treated with aspirin or clopidogrel was similar to that in patients with symptomatic severe ICAS treated with aspirin or warfarin in the WASID study [6], but was higher than that in patients of the medical arm of SAMMPRIS trial [11], which may be due to the facts that aggressive medical management (dual antiplatelet therapy in the first 90 days followed by aspirin alone, intensive management of vascular risk factors, and a lifestyle-modification program) used in the SAMMPRIS trial was not used to the same extent in our patients. We also found that a reduced MCA branch signal intensity was associated with internal border-zone infarction and that a severely reduced signal intensity of the MCA branches was a strong predictor of future stroke in the ipsilateral MCA in patients with severe MCA trunk stenosis. Moreover, studies with a long study period should be performed to evaluate the implications of MCA branch signal intensity distal to the site of stenosis for the long-term prognoses in patients with severe MCA trunk stenosis.

A previous study graded the signal intensity of the MCA ipsilateral to ICA stenosis according to the extent to which the MCA could be visualized on MRA; this approach resulted in good interobserver and intraobserver agreement; the signal intensity of the MCA was visually classified into 4 grades: all M3 branches of the MCA could be visualized to the cortical surface (Grade A); one or more M3 branches could not be visualized to the cortical surface (Grade B); one or more M2 branches could not be visualized along its course (Grade C); and the M1 could not be visualized along its course (Grade D) [25]. Although this study’s method of grading the signal intensity of the MCA branches on MRA was adapted from the above method, we made several modifications to improve its diagnostic strength. First, we excluded patients with contralateral MCA or ICA severe stenosis or occlusion and compared the signal intensities of the MCA branches with those of the contralateral MCA branches in all patients to reduce the influence of individual differences in cerebral blood flow velocity or the number of MCA branches. Second, we strengthened the parameters of the grade B classification.

In this study, for severe MCA trunk stenosis causing ischemic stroke or TIA, the risk of recurrence in the ipsilateral MCA in the patients with severely reduced signal intensity of the MCA branches was higher than that in the patients with no or mildly reduced signal intensity. Additionally, a severely reduced signal intensity of MCA branches was a strong predictor of stroke recurrence. For 3D TOF MRA, the degree of flow signal reduction distal to the site of stenosis depended on the severity of the stenosis, and the reduction of flow signal intensity indicated impaired flow and perfusion distal to the stenosis [19–21, 25]. In a study comparing the signal intensity of the MCA on 3D TOF MRA versus the cerebrovascular reactivity quantified via perfusion single-photon emission CT in patients with cervical ICA steno-occlusive diseases, the reduced signal intensity of the MCA on MRA could be used to assess the hemodynamic impairment with 86.2 % sensitivity and 69.6 % specificity [25]. Our study demonstrated that the reduced signal intensity of the branch arteries distal to the MCA stenosis was associated with internal border-zone infarction. We speculated that a severely reduced signal intensity of MCA branches may be related to a more severe narrowing of the MCA trunk lumen and severe perfusion impairment, which may explain the higher risk of recurrence.

The findings of our study may have important implications. Because a severely reduced signal intensity of the MCA branches on MRA was a strong predictor of stroke recurrence in the ipsilateral MCA in patients with severe MCA trunk stenosis in this study and may indicate severely impaired flow and perfusion [19–21, 25], we propose that aggressive treatments (anticoagulant or endovascular revascularization) may reduce the risk of stroke recurrence in patients with severe MCA stenosis causing ischemic stroke or TIA when a severely reduced signal intensity of the ipsilateral MCA branches is observed via MRA. However, the above hypothesis needs to be verified in future studies.

This study was the first study to evaluate the relationship between the signal intensity of MCA branches on MRA and the risk of stroke recurrence in patients with ischemic stroke or TIA caused by severe MCA stenosis. One major advantage of this study was the use of MRA for grading the signal intensity of the MCA branches distal to the MCA trunk stenosis. This method may have good prospects for clinical use because it is easy for clinicians to implement and exhibits good interobserver and intraobserver agreement.

This study had several limitations. First, if the MCA stenosis chronically progresses, the collateral blood flows via leptomeningeal anastomosis often perfuse the area distal to stenosis, and hemodynamics may remain normal. As a result of higher saturation of inflowing spins due to longer paths of collateral blood flow, collateral blood flow signals are lost, and the affected MCA branches would not showed on single-slab 3D TOF MRA. The relationship between the signal intensity of the branch arteries distal to MCA stenosis on MRA and cerebral perfusion should be investigated in future studies. Second, when one or more MCA branches do not appear on MRA, they may simply be occluded, and the grade of visualization may not reflect the velocity of blood flow distal to the MCA stenosis. Third, it was difficult to obtain an accurate stenosis rate on the condition that the severe stenosis of MCA trunk presented with a focal signal loss with presence of distal MCA signal on MRA. So, for severe MCA trunk stenosis, the relationship between the level of stenosis rate and the flow signal intensity of the branch arteries distal to the stenosis on MRA should be investigated in future studies using both DSA and MRA to assess the cerebral arteries, which may be beneficial for understanding the mechanisms underlying the signal reduction of the MCA branches distal to the stenosis on MRA. Fourth, this single-center study set the inclusion criteria of patients with ischemic stroke or TIA within 7 days of stroke onset in order to achieve a large sample size, because cranial MRA was performed within 7 days after onset in almost all patients with ischemic stroke or TIA at our center. However, although 7 days may be regarded as a short period, the changes in hemodynamic factors (such as blood pressure and the capability of collateral circulation) may result in changes in the signal intensity of the MCA branches distal to the stenosis on MRA, which should be investigated further in future studies. Fifth, as the discontinuation of antiplatelet drug or statin during the follow-up period was observed in a small number of cases and was not associated with ipsilateral stroke recurrent, and no significant differences in the discontinuation of antiplatelet drug or statin were observed between patients of different groups in this study; the impact of the discontinuation of these drugs on the incidence of stroke recurrence in all patients or patients of different groups may be small.

## Conclusions

In conclusion, our study demonstrated that the signal intensity of the branch arteries distal to the MCA stenosis may be a useful and simple approach to stratify the risk of stroke recurrence in patients with ischemic stroke or TIA caused by severe MCA stenosis. The findings of this study should be considered in future studies exploring the treatment of ICAS.

## Abbreviations

ICAS: Intracranial artery stenosis
TIA: Transient ischemic attack
SAMMPRIS: Stenting and aggressive medical management for preventing recurrent stroke in intracranial stenosis
WASID: Warfarin aspirin symptomatic intracranial disease
MRA: Magnetic resonance angiography
3D: Three-dimensional
TOF: Time-of-flight
SIR: Signal intensity ratio
ICA: Internal carotid artery
CEA: Carotid endarterectomy
DWI: Diffusion-weighted imaging
TR: Repetition time
TE: Echo time
HR: Hazard ratio
CI: Confidence interval
